# Fructooligosaccharides for Relieving Functional Constipation: A Systematic Review and Meta-Analysis of Randomized Controlled Trials

**DOI:** 10.3390/foods13243993

**Published:** 2024-12-11

**Authors:** Hongmin Zhen, Hanwei Qian, Xiaoyan Liu, Chen Tan

**Affiliations:** 1 Key Laboratory of Geriatric Nutrition and Health, Ministry of Education, China-Canada Joint Lab of Food Nutrition and Health, Beijing Technology and Business University (BTBU), Beijing 100048, China; zhenhongmin@btbu.edu.cn (H.Z.); 2230202133@st.btbu.edu.cn (H.Q.); 2 China Food Flavor and Nutrition Health Innovation Center, School of Food and Health, Beijing Technology and Business University, Beijing 100048, China

**Keywords:** fructooligosaccharides (FOS), constipation, meta-analysis, gut microbiota

## Abstract

Fructooligosaccharides (FOS) are prebiotics with great potential to improve constipation. This study set out to investigate the usefulness of consuming FOS as a dietary supplement on bowel movement frequency, stool consistency, abundance of Bifidobacteria, gastrointestinal transit time, and gastrointestinal symptoms through a systematic review and meta-analysis of randomized controlled trials (RCTs). We searched the Cochrane Library, PubMed, EMBASE, and Web of Science databases up to 2 March 2024. Randomized controlled trials reporting the use of FOS as a supplement by participants were included. Bias risk was assessed using the Cochrane Risk of Bias assessment tool. Results were synthesized using a random-effects model or fixed-effects model, combining outcomes using odds ratios (OR), weighted mean differences (MD), or standardized mean differences (SMD) with 95% confidence intervals (CI). The meta-analysis encompassed 17 randomized controlled trials, involving a total of 713 study participants. Overall, the intake of FOS significantly increased bowel movement frequency (95% CI: 0.80, 1.50, *p* < 0.00001) and positively affected stool consistency, softening the stool (SMD: 0.36, 95% CI: 0.12, 0.60, *p* = 0.76). It also reduced the effort and pain during defecation (SMD: −0.60, 95% CI: −0.85, −0.34, *p* = 0.12). At the same time, mild bloating was noted as an adverse event associated with FOS ingestion (OR: 10.36, 95% CI: 3.32, 33.23, *p* = 0.24). No reports of serious adverse events were documented. Overall, FOS may enhance bowel movement frequency, stool consistency, and overall constipation symptoms, suggesting cautious optimism regarding their use as a dietary treatment alternative. Nevertheless, further robust and definitive randomized controlled trials are required to more accurately determine the most effective dosage and duration of use. Additional research and evidence are necessary before the efficacy of FOS as a therapeutic method for treating functional constipation can be firmly established.

## 1. Introduction

Constipation is a digestive disorder caused by prolonged retention of feces in the colon, resulting in difficulty in defecation, lower frequency of defecation, or dry, hard feces, frequently coexisted by manifestations of bulking and abdominal pain, which may be principal (idiopathic or functional) or secondary to other factors, such as medication side-effects (opioids) or disease [1]. It is partly related to disorders in gut-brain interaction, and suffering from neurological disorders are at an elevated threat of developing fecal incontinence and constipation [2]. Furthermore, constipation is also related to changes in the colonic microbiota, diet, and other lifestyle factors such as colonic motility, as well as psychological factors [3,4,5].

Functional constipation (FC) is the most prevalent kind of constipation, occurring across the entire age spectrum, ranging from infancy to the elderly [6]. The global incidence of FC is about 10.1% in adults, with higher rates observed in countries such as France, Italy, and Japan [7]. Additionally, the incidence of constipation is significantly high among women and the aging population. According to a study assessing the worldwide frequency of constipation in the elderly people, the occurrence of constipation among 58,405 seniors between the ages of 60 and 93 was 18.9% overall (95% CI: 14.7–23.9) [8]. Lasting FC not only had significant impact on the health and quality of life, but also increased the chance of several other diseases, resulting higher medical burden [9]. If chronic constipation is not addressed quickly, it can lead to more serious disorders such fecal impaction, incontinence, and bowel perforations, which would further increase healthcare expenses and patients’ health-related quality of life (HR-QOL) [10]. Analysis of HR-QOL data from 2344 children showed that children with functional constipation had lower overall HR-QOL total scores compared to healthy children [11]. The main strategies for constipation treatment include dietary modification, medications such as laxatives, and anorectal biofeedback [7,12,13]. However, the high costs and adverse side effects of these medications limit their use in managing constipation. Excitatory laxatives can cause structural damage to the epithelial cell surface, while certain irritant laxatives may harm intestinal nerves and are associated with increased risks of coronary heart disease (CHD) and ischemic stroke [14]. Other common side effects include nausea, diarrhea, and abdominal pain. Therefore, dietary modifications are pivotal in the treatment of constipation, which also are sufficient to alleviate the symptoms of constipation [15].

In 2017, the definition of a prebiotic has been modified to ‘a substrate that is selectively utilized by host microorganisms conferring a health benefit’ by the International Scientific Association for Probiotics and Prebiotics (ISAPP) consensus statement. Prebiotics primarily consist of functional oligosaccharides, dietary fiber, resistant starch, and other undigested carbohydrates [16]. Fructooligosaccharides (FOS) consist of a mixture of sucrose trisaccharides, sucrose tetrasaccharides, and sucrose pentasaccharides formed by combining one to four fructose units with sucrose through a β(2-1) glycosidic bond [17]. In fact, as one of the most popular commercial prebiotics, FOS can be preferentially degraded by bifidobacteria due to the prevalent β-fructanosidase in bifidobacteria [16]. The products of bacterial fermentation of FOS in the colon are short-chain fatty acids (SCFAs), which are absorbed and utilized by colonic epithelial cells to stimulate cell growth and enhance the absorption of salt and water [18]. This process increases moisture in the cecal bolus through osmotic pressure, leading to peristalsis and relieving constipation [19]. To date, only one prebiotic chicory inulin was approved by the European Food Safety Authority (EFSA) to get an EU health claim, “Inulin improves bowel function”, attributed to the cause-effect relationship between consumption of chicory inulin and maintenance of normal defecation by increasing stool frequency [20]. Patients with constipation are usually advised to increase their dietary fiber intake.

The potential of FOS supplementation in relieving constipation is notable. Previous systematic reviews have assessed the effects of prebiotics or dietary fiber on intestinal function; however, few meta-analyses have specifically explored the effects of FOS on constipation, and its mechanism of action remains inadequately detailed, posing challenges to its clinical application. Therefore, we aimed to investigate the effects of FOS as a dietary supplement on the frequency of defecation, fecal consistency, Bifidobacteria abundance, intestinal transit time, and gastrointestinal symptoms in participants, with the hope of gaining further insights into the role of FOS in alleviating constipation.

## 2. Materials and Methods

We conducted a systematic review and a meta-analysis to evaluate the effect of FOS on bowel function. The primary outcomes were the frequency of bowel movements and stool consistency, as assessed by the number of bowel movements per week during the study period. Secondary outcomes included bifidobacterium genus counts, straining effort and pain, abdominal distension, and gut transit time. The present review was conducted in accordance with the Preferred Reporting Items for Systematic Reviews and Meta-Analyses (PRISMA) guidelines.

### 2.1. Eligibility Criteria

The inclusion criteria for the study are as follows: (1) The study must be a randomized controlled trial; (2) Participants of various ages and health conditions are eligible; (3) The intervention must include fructo-oligosaccharides; (4) The intervention period should be at least seven days; (5) The study must report one or more of the following outcomes: bowel frequency, stool consistency, abundance of Bifidobacteria, intestinal transit time, and overall assessment of gastrointestinal symptoms; (6) The publication must be in English.

### 2.2. Literature Search

A comprehensive literature search using PubMed, Web of Science, Embase, and Cochrane Library was performed to identify intervention studies in human populations up to 2 March 2024 (the end date of the digital search process). The full search string used in each database is: (fructooligosaccharides OR fructo-oligosaccharides OR fructooligosaccharides OR fructans) AND (constipation OR functional constipation OR dyschezia OR idiopathic constipation OR slow transit OR colonic inertia) NOT animal.

The supplementary literature search involved a detailed examination of the reference lists of all relevant trials and key reviews, with the aim of identifying and collecting important articles that had been overlooked in the initial search.

### 2.3. Study Selection

Two authors independently selected studies based on predefined inclusion criteria using the online software EndNote X9, which screened titles and abstracts and then retrieved and assessed the full text of potentially relevant studies for final inclusion. Any disagreements were resolved through discussion and consensus.

### 2.4. Data Extraction

All of these meta-analyses were conducted using the Review Manager software 5.4.1. Data were extracted from each eligible study for the following items: name of first author, publication year, study origin (country), the type of chicory-derived inulin-type fructan intervention (fructooligosaccharides, mixture of FOS), dosage and duration of the intervention, health status of participants, sex, age group (infants < 1 year; children 1–18 years; adults 18–65 years; and senior adults > 65 years), sample size, stool frequency (number/week), stool consisitency, abundance of Bifidobacterium, gut transit time, health related quality of life parameters. To facilitate comparison between studies, different units of measurement have been converted into a standardized form.

### 2.5. Data Synthesis and Statistical Analysis

Dichotomous data were analyzed using odds ratio (OR), while continuous variable data were analyzed using mean difference (MD) or standardized mean difference (SMD). Heterogeneity between studies was assessed using the chi-square-based Q statistical test, with *p*-values and I^2^ statistic ranging from 0% to 100% to quantify the extent of heterogeneity. A *p*-value ≤ 0.10 was considered indicative of significant heterogeneity and pooled risk ratios (RRs) were estimated using a random effects model. Conversely, if statistical study heterogeneity was not observed (*p* > 0.10), a fixed-effects model was employed.

### 2.6. Quality Assessment and Risk of Bias

The risk of bias was assessed by two independent reviewers. The Cochrane Risk of Bias assessment tool, recommended by the Cochrane Handbook, evaluates bias risk in six domains: selection bias, performance bias, detection bias, attrition bias, reporting bias, and other biases. Judgements were categorized as “low risk”, “unclear risk”, or “high risk” and were visually represented using figures generated by RevMan5.4 software.

## 3. Results

### 3.1. Study Inclusion

**Figure 1:**
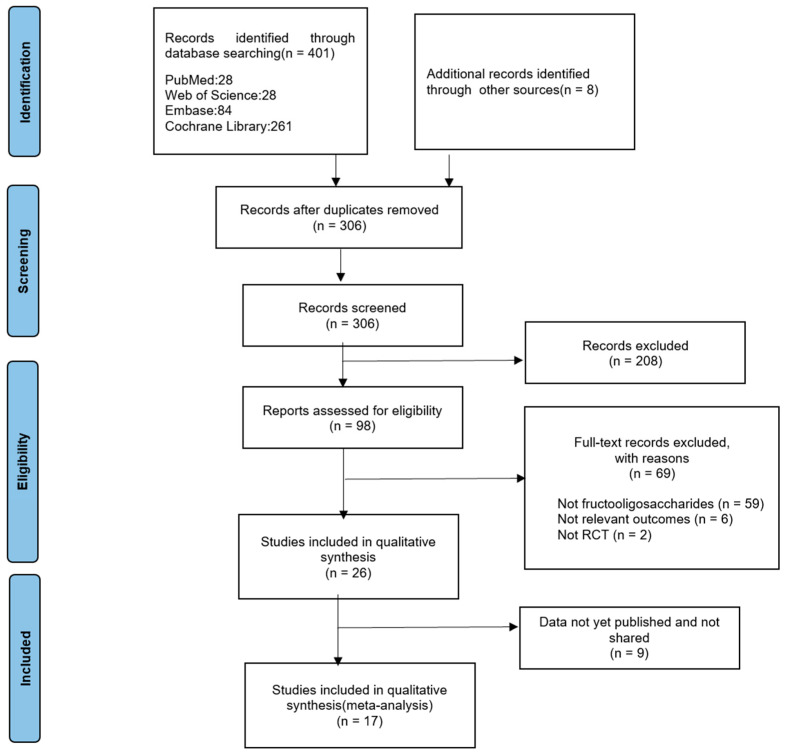
PRISMA Flowchart of study selection.

The literature search process is illustrated in Figure 1. A total of 401 articles were retrieved from PubMed, Embase, Web of Science, and the Cochrane Library. By examining the references in these articles, eight additional papers were identified. After removing duplicate entries and screening the remaining articles based on their titles and abstracts, a total of 208 papers were included. Upon full-text examination, 69 records were excluded for various reasons, including interventions not involving FOS (59), lack of relevant outcomes (11), non-randomized controlled trials (2), and unpublished or unshared data (9). Ultimately, 17 papers met the eligibility criteria for inclusion in the analysis.

### 3.2. Study Characteristics

The characteristics of the included studies are presented in Table 1, highlighting studies conducted between 1999 and 2024. Out of the 17 trials, 10 studies used single FOS as the intervention [21,22,23,24,25,26,27,28,29,30]. Additionally, one study used a mixture of polydextrose (PDX) and FOS, one study used yacon syrup rich in FOS [31], one study employed wheat bran extract (WBE) containing xylooligosaccharides and FOS [32], and one study involved agave fructans, a mixture of FOS and fructans [33]. Furthermore, three studies used a mixture of inulin and FOS [34,35,36]. Participants in 4 trials were infants and children aged between 0 and 18 years, while participants in 12 studies were adults, with 1 study not explicitly reporting the age range. Six studies included healthy participants, while the remaining studies involved participants with constipation-related symptoms.

### 3.3. Primary Outcomes

#### 3.3.1. Stool Frequency

**Figure 2:**
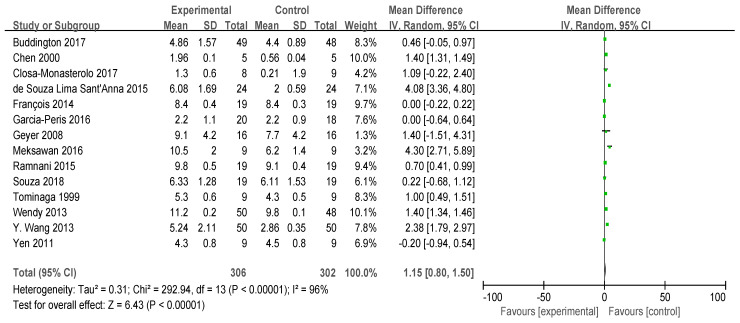
Forest plot for the effect of FOS on stool frequency [21,22,23,25,26,28,29,30,31,32,33,34,35].

Sixteen studies recorded bowel movement frequency, although two did not provide standard deviation (SD) values. Consequently, a meta-analysis was conducted on 14 studies (Figure 2), involving a total of 608 participants (N_exprimental_ = 306, N_control_ = 302). Overall, FOS intake significantly increased bowel frequency compared with controls, but there was considerable heterogeneity (I^2^ = 96%, *p* < 0.00001). The effect of FOS on bowel movement frequency may vary among individuals; some individuals may experience an increase in bowel movement frequency after consuming FOS. However, other individuals may not exhibit a significant increase in bowel movement frequency following FOS consumption, potentially due to their lower sensitivity to FOS.

**Figure 3:**
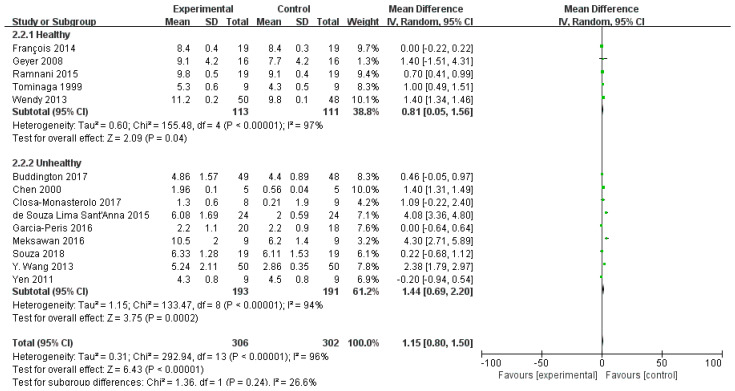
Subgroup analysis of stool frequency after FOS intake based on health status [21,22,23,25,26,28,29,30,31,32,33,34,35].

We conducted subgroup analyses of stool frequency in both healthy and unhealthy participants (Figure 3). The supplementation of FOS is beneficial for both healthy and unhealthy volunteers to increase the frequency of bowel movements, but a considerable heterogeneity was detected (*p* < 0.00001, I^2^ = 97%; *p* < 0.00001, I^2^ = 94%;). This variability may be attributed to differences in study methodologies, interventions, or diverse health and physiological conditions of the participants. Therefore, additional experimental studies with more appropriate designs are needed to achieve high certainty of evidence.

#### 3.3.2. Stool Consistency

**Figure 4:**
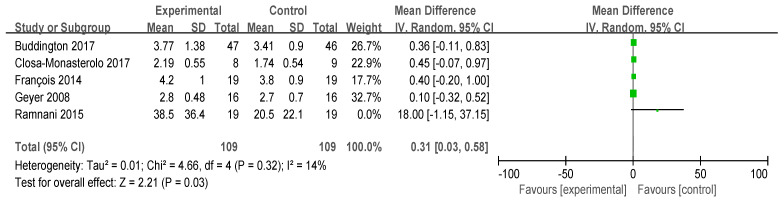
Forest plot for the effect of FOS on stool consistency [21,31,32,33,34].

Five studies reported on stool consistency (Figure 4), involving a total of 218 participants (N_exprimental_ = 109, N_control_ = 109). The methods used to measure stool consistency vary across studies, including the Bristol Stool Scale or its modified versions, thus standardized mean difference (SMD) values were calculated. No statistical heterogeneity was observed (*p* > 0.10), allowing for the use of a fixed-effect model. Overall, FOS has a significant softening effect on stool consistency (I^2^ = 14%, *p* = 0.32). One study reported that FOS supplementation more than doubled the wet weight of each stool compared to the control group [22].

### 3.4. Secondary Outcomes

#### 3.4.1. Bifidobacterium Genus Counts

Four studies reported changes in the quantity of Bifidobacteria; however, two of them did not provide standard deviation values, resulting in insufficient data for a meta-analysis. Nevertheless, all these studies demonstrated an increase in the quantity of Bifidobacteria after consuming FOS. One study indicated that the intake of FOS by infants with constipation results in an increase in the abundance of Bifidobacterium in their fecal samples [26]. Moreover, a recent study proved that short-chain GOS and FOS (degrees of polymerization (DP) 2 and 3) were favored growth substrates for strains of *B. bifidum* and *B. longum* subsp. longum, whereas both *B. breve* and *B. longum* subsp. infantis had the ability to utilize both short- and longer-chain GOS and FOS (DP 2 to 6) [38].

#### 3.4.2. Straining Effort and Pain

**Figure 5:**
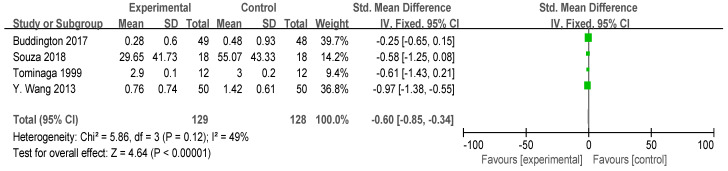
Forest plot for the effect of FOS on straining effort and pain [21,26,28,29].

Four studies reported the effort and pain scores during defecation (Figure 5), encompassing a total of 257 participants (N_exprimental_ = 129, N_control_ = 128). Due to variations in the methods used to assess and measure gastrointestinal sensation, the Standardized Mean Difference (SMD) was calculated. No statistical heterogeneity was observed (*p* > 0.10), allowing for the application of a fixed-effect model. The intake of FOS significantly reduced the effort and pain during defecation (I^2^ = 49%, *p* = 0.12), which may be related to the softening effect of FOS on feces. While most doctors consider infrequent bowel movements as a sign of constipation, most patients believe that excessive straining is the most crucial aspect of constipation [39].

#### 3.4.3. Abdominal Distension

**Figure 6:**
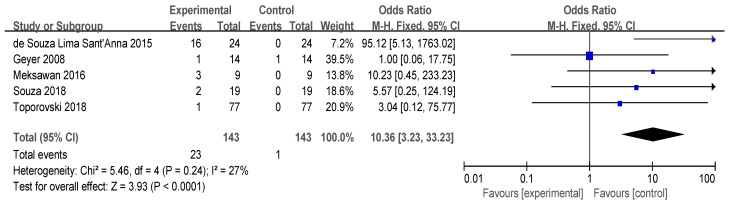
Forest plot for the effect of FOS on abdominal distension [25,26,31,35,37].

Seven studies reported that the intake of FOS may lead to bloating and flatulence. However, two studies did not specify the exact number of participants, so a total of five studies were included in the meta-analysis (Figure 6). No statistical heterogeneity was observed (*p* > 0.10), permitting the use of a fixed-effect model. The results indicated that consuming FOS can result in adverse effects such as flatulence and bloating (I^2^ = 27%, *p* = 0.24); however, all studies indicated that these adverse effects were within tolerable limits. As a prebiotic, FOS is utilized by the gut microbiota, leading to gas production during fermentation, which may cause bloating.

#### 3.4.4. Gut Transit Time

Four studies reported that the intake of FOS accelerates intestinal transit. However, due to incomplete data, a meta-analysis could not be conducted. FOS may accelerate intestinal transit by forming a gel-like substance after absorbing water in the intestines, thereby increasing the volume and viscosity of intestinal contents. This process stimulates intestinal motility, promoting the speed of food transit in the intestines. This effect may lead to an increase in defecation frequency, thus helping to improve gastrointestinal issues such as constipation.

#### 3.4.5. Individual Gastrointestinal Symptoms

One study demonstrated that the percentage of children experiencing abdominal pain symptoms decreased significantly from 84.2% to 2.6% after consuming FOS. However, it is important to note that there was no placebo-treated control group in this study. Another study indicated that, compared to the control period, participants experienced a significant decrease in “noise”, “pressure”, and “pain” values after consuming FOS, especially at higher doses.

### 3.5. Quality of Evidence and Risk of Bias in Studies

**Figure 7:**
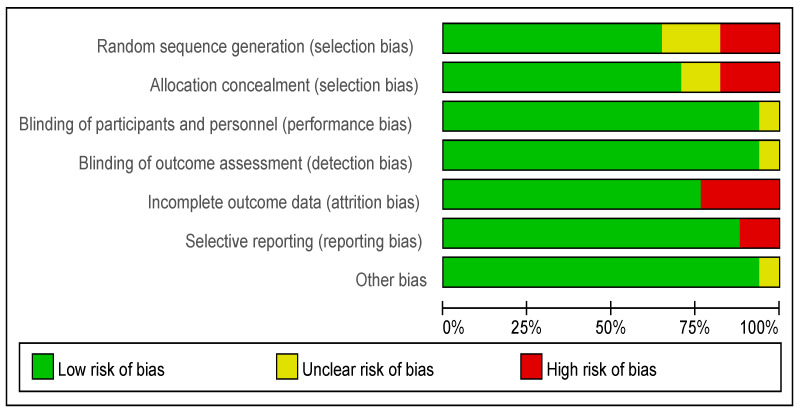
Risk of bias graph: the judgements about each risk of bias item presented as percentages across all included studies.

**Figure 8:**
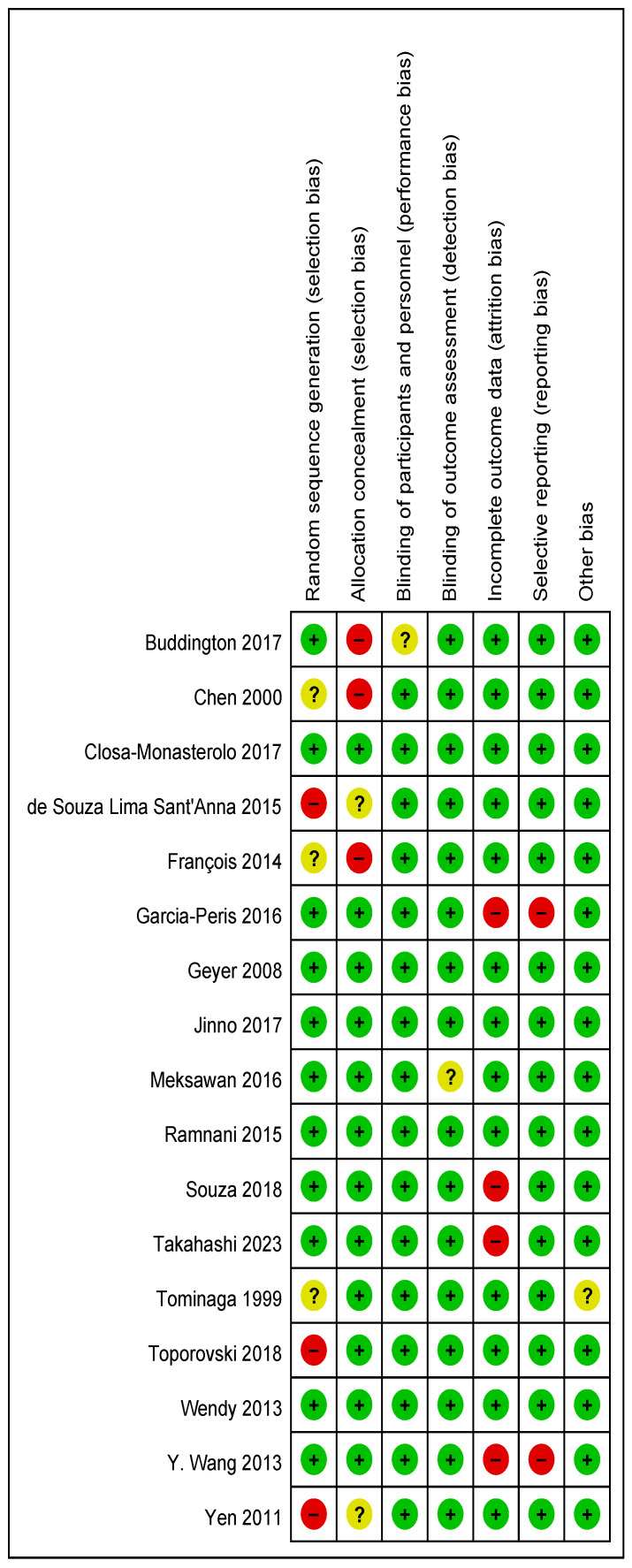
Risk of bias summary: the judgements about each risk of bias item for each included study [21,22,23,24,25,26,27,28,29,30,31,32,33,34,35,36,37].

The risk of bias for each randomized controlled trial was assessed across all included studies and separately for each study (Figure 7 and Figure 8). The risk of bias analyses indicated generally good methodological quality. All randomized controlled trials demonstrated a low risk of bias for performance bias, detection bias, and other biases, with an unclear risk of bias primarily for selection bias. The high risk of bias was mainly attributed to selection bias, attrition bias, and reporting bias.

## 4. Discussion

**Figure 9:**
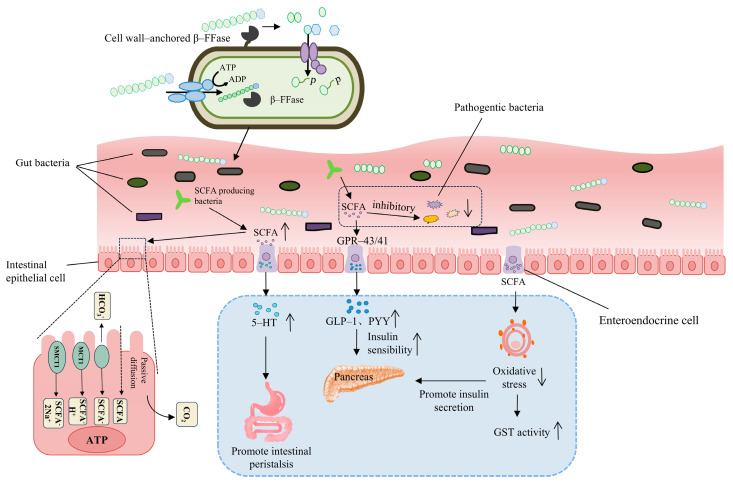
Putative, microbe-driven mechanisms for FOS action and the functional roles of SCFAs. FOS is fully transported and hydrolyzed by cytoplasmic GH32 family β-fructofuranosidase. The extracellular hydrolysis of FOS is mediated by surface-associated GH32 β-fructofuranosidase, followed by the uptake of hydrolysis products through one or more transport proteins. SCFAs are rapidly absorbed by colonic cells via monocarboxylate transporters, passive diffusion, or an exchange mechanism with bicarbonate (HCO_3_^−^), subsequently undergoing partial oxidation to carbon dioxide (CO_2_) to generate ATP for cellular energy. SCFAs can enhance intestinal motility by activating receptors that promote the production of 5-hydroxytryptamine (5-HT), leading to increased synthesis and release of 5-HT. Additionally, SCFAs can activate GPR-43 and GPR-41 receptors, resulting in the production of gut peptides such as GLP-1 and PYY, which enhance pancreatic sensitivity to insulin. SCFAs exhibit antioxidant activity, reducing intracellular oxidative stress and protecting cells from oxidative damage, thereby mitigating β-cell injury and promoting insulin secretion. By decreasing oxidative stress, SCFAs also help maintain the activity of glutathione S-transferase (GST), thereby enhancing its capacity to effectively eliminate harmful substances.

The abundance of bifidobacteria may influence bowel frequency and fecal consistency. This effect may be attributed to the ability of *Bifidobacterium* to break down cellulose and other indigestible substances in food, which increases the water content in the stool, facilitates easier passage through the intestines, and results in softer stools [40]. One study showed that in children with constipation and abdominal pain, the intake of FOS significantly improved the symptoms [37]. Prebiotics can selectively enhance the activity of specific groups of colonic bacteria. However, a study employing six strains of probiotic bifidobacteria (*Bifidobacterium breve*, *Bifidobacterium lactis*, *Bifidobacterium longum*, *B. breve CCUG 24611*, *B. lactis JCM 10602*, and *Bifidobacterium pseudocatenulatum* JCM 1200) proved that all these bifidobacteria strains exhibited extracellular antimicrobial activity against four *Clostridium difficile* strains, including the CD NAP1/027. Moreover, none of the *C. difficile* strains degraded five prebiotic-non-digestible oligosaccharides including FOS [41]. Changes in microbial composition affect gut function and metabolite production [42], whereas certain metabolites affect gut motility (Figure 9). For example, the fermentation of dietary fibers into short-chain fatty acids (SCFAs) by the gut microbiota, which can promote intestinal peristalsis by activating receptors in 5-hydroxytryptamine (5-HT)-producing cells and increasing the synthesis and release of 5-HT [43]. Disruption of intestinal microbiota affects SCFA production, which reduces glucagon-like peptide-1 (GLP-1) production and subsequently inhibits intestinal transmitter function [44]. SCFAs play a crucial role in gut health and have extra-intestinal metabolic effects beyond the intestines, such as regulating glucose homeostasis, lipid metabolism, immune function, and appetite (Figure 9) [45]. After feeding mice different doses of FOS for four weeks, it was observed that the high-dose group had higher concentrations of SCFA compared to the low-dose and the control groups. It was also discovered that FOS predominantly stimulates the growth of *Bifidobacterium pseudolongum* in mice [46]. In recent years, research on intestinal diseases and the gut microbiome has gradually revealed the connection between constipation and disorders in intestinal flora, providing a theoretical foundation for microbiological treatment of chronic constipation.

Typically, FOS with a DP ranging from 2 to 9 are more fermentable molecules, particularly utilized by Bifidobacteria and Lactobacillus [18,47]. A meta-analysis revealed that consistent consumption of β-fructans significantly increased the frequency of bowel movements in populations with and without intestinal disorders. This effect was attributed to short-chain β-fructans, rather than long-chain β-fructans (DP ≥ 10) [48]. In certain bacteria, long-chain FOS require the action of exogenous enzymes before being utilized [49]. However, this type of enzyme is rare in Bifidobacteria, indicating a preference for short-chain FOS substrates. The regulatory mechanism of low-FOS utilization in Bifidobacteria remains unclear, particularly regarding the transport system involved in FOS absorption. In species of Lactobacillus that metabolize FOS, FOS utilization may proceed through one of the following two metabolic pathways: (a) complete substrate transport and hydrolysis by cytoplasmic GH32 family β-FFase; (b) extracellular hydrolysis of the substrate by cell surface-associated GH32 β-FFase, followed by uptake of the hydrolysis products by one or more transport proteins. Most Bifidobacteria and Lactobacillus species that utilize FOS have dedicated transport proteins and intracellular β-FFase for the breakdown of primarily low-DP FOS substrates. The cscA gene encoding β-FFase has been identified in *Bifidobacterium adolescentis*, *B. breve*, and *B. longum* [50]. Interestingly, CscA hydrolyzed the β-2,1 linkage between the glucose and fructose moieties of FOS but not the β-2,1 linkage between two fructose moieties within the same substrate, leaving behind chains of fructose molecules as residual hydrolytic products (Figure 9).

In addition, FOS also contribute to the expression of calcium-binding proteins, enhancing the absorption of certain minerals (such as calcium) and regulating the expression of cytokines in the bones [51]. Research has found that FOS can regulate lipid metabolism, reducing the risk of developing diabetes and obesity [19]. A diet high in animal fats and low in dietary fiber concentration increases the risk of developing colon cancer. Research has shown that FOS can inhibit the development of colon cancer by increasing the levels of beneficial bacteria and SCFAs produced during the fermentation of FOS in the colon. This promotes the growth of healthy bacteria that suppress the growth of pathogens, thereby reducing the production of carcinogens and bacteria enzymes involved in the carcinogenic process in the colon [52]. Linear fructans with low DP and branched chains fructans have a high antioxidant capacity due to SCFAs, which reduce oxidative stress within cells and protect them from oxidative damage. By reducing oxidative stress, SCFAs may play a role in maintaining GST activity, allowing for more effective scavenging of harmful substances [53]. Consuming FOS also has beneficial effects on the immune modulation of the intestinal immune system [17,54].

An increase in the frequency of bowel movements is a beneficial physiological effect of fiber as long as it does not lead to diarrhea. FODMAP represents fermentable oligosaccharides, disaccharides, monosaccharides, and polyols, which are short-chain carbohydrates that are poorly absorbed by the small intestine [55]. Due to much of the existing research suggesting that negative side effects of consumption of FODMAP are common, the low FODMAP diet serves as a second-line dietary intervention for patients with irritable bowel syndrome (IBS) [56]. However, the intake of FOS was not reported to cause diarrhea in any of the trials included in this review, which is attributed to the difference of health and physiological conditions of participants in this meta-analysis. FOS is generally considered to be well tolerated, but targeted assessment of negative intestinal events is still required, which is often lacking in nutrition or supplement-based trials. A study indicates that the maximum daily intake should not exceed 30 g [57], which is a challenging dose for most people to reach the concerning the recommended daily intake of dietary fiber [58]. Some individuals may experience a higher frequency of mild and transient gastrointestinal symptoms when ingesting more than 10–12 g/day of FOS, particularly when taken occasionally [59]. In a dose-response study of FOS supplementation in healthy volunteers, a dose of 10 g/day was observed to be the minimum dose capable of inducing bifidogenic effects [60,61]. Significant individual variation is recognized in response to the fermentation of prebiotics, and the stoichiometry of fermentation likely varies according to carbohydrate chain length and monosaccharide composition. These observations are consistently supported by in vitro fermentation studies, which indicate that longer-chain molecules undergo fermentation at a slower rate and produce less net hydrogen excretion. The development of prebiotics with diverse chain lengths, degrees of branching, and DP may decrease gas production and positively modify their properties by selectively influencing the microbial community, thereby contributing to overall health of host [62].

To date, no meta-analysis has been conducted to evaluate the effect of FOS on the relief of constipation. In the present study, we synthesized data from 17 independent studies for meta-analysis, assessing various outcomes, including stool frequency, stool consistency, counts of the *Bifidobacterium* genus, straining effort and pain, abdominal distension, gut transit time, and individual gastrointestinal symptoms, to examine the effect of FOS on constipation. These results align with previous recommendations regarding the consumption of FOS to aid in the treatment of constipation, suggesting that FOS intake may be considered a secondary preventive measure for patients with constipation. However, the results of this meta-analysis show that FOS alleviate constipation but are frequently associated with bloating, highlighting the need for accurate dosing in the practical application of FOS for constipation relief. The presence of heterogeneity in this meta-analysis necessitated careful consideration, as it influenced the interpretation and applicability of our findings. A likely source of this heterogeneity was the variability in study characteristics across the included publications. Factors such as differences in study design, patient demographics, and intervention protocols may contribute to the observed heterogeneity. Furthermore, variations in the quality of evidence within the included studies, including differences in study quality, methodology, and risk of bias, may also contribute to heterogeneity. Also, the limited number of experimental studies of the effects of FOS on constipation and the limited number of targeted participant populations restricts the availability of sufficient data for subgroup analyses. Given the complexity of differences among studies, it is indicated that more research is needed to clarify the role of FOS in the prevention of constipation in participants with different ages and physiological conditions.

## 5. Conclusions

In conclusion, FOS can enhance bowel movement frequency, improve stool consistency, and increase the abundance of *Bifidobacterium*. As a viable alternative for alleviating constipation, particularly short-chain FOS with a DP less than 10 may offer an effective option compared to conventional treatments such as laxatives. However, to reduce the incidence of adverse events, such as bloating, further experimental research with larger populations and more appropriate designs are necessary to identify the optimal dosages of FOS supplements for managing constipation.

## Figures and Tables

**Table 1:** Summary of all studies included in this paper.

Study, Year (Ref.)	Country	Health Status	Gender	Age	Sample Size	Control Used	Intervention	Dose	Form	Duration	Outcomes Included in Meta-Analysis	Primary Outcome
Chi [30]	China taiwan	Constipation (>6 month)	Both	74 ± 3.3	9	Placebo (fructose syrup)	FOS	10 g/d	drink	8 wk	Stool frequency; Bifidobacterium genus counts	Increased dry fecal mass; Daily fecal output of bifidobacteria significantly increased with FOS
[25]	Thailand	CAPD patients; chronic constipation	Both	>50	9	Placebo (sucrose)	FOS	20 g/d	approximately 150–200 mL of water	30 d	Stool frequency; Abdominal distension; Colonic transit time	FOS significantly increased the frequency of defecation; The colonic transit was augmented; Mild discomforts
[29]	China	Constipation	Both	>18 56.6 ± 14.3	100	Placebo (starch)	FOS capsules	1260 mg/d	capsules	10 d	Stool frequency; Scores of straining effort and pain	FOS significantly increased the bowel evacuation rate, reduced perception of straining effort and pain, and improved the quality of stools.
[21]	Germany	body mass index ≤ 35; 1 to 3 reported bowel movements per week	Both	18–65 34.2 ± 12.1	97	Placebo (maltodextrin)	Oligofructose	0~15 g/d	dissolved in a drink	12 wk	Stool frequency; Stool consistency; Straining effort and pain	Stool frequency did not increase significantly; Stool consistency remained unchanged; Values for “noise”, “pressure”, and “pain” significantly decreased
[35]	Brazil	intestinal constipation	Both	20–75 39.77 ± 17.81	48	Placebo (maltodextrin)	mixture of inulin and FOS	10 g/d	orange juice	30 d	Stool frequency; Straining effort and pain; Abdominal distension;	Increased number of evacuations; Improvement in the consistency of the feces; the group that consumed the YBP showed higher counts of Bifidobacterium; Tolerable symptoms of flatulence were reported by 13 volunteers.
[37]	Brazil	constipation for at least 1 month prior	Both	4–8	77	(regular diet)	mixture of PDX and FOS	PDX 4.17 g and FOS 0.45 g	food supplement	45 d	Abdominal distension	Significant reduction in the frequency of constipation symptoms of the children evaluated. The tolerability was very good, and the rate of adverse effects was low.
[26]	Brazil	Constipation	Both	6–24 months	36	Placebo (maltodextrin)	FOS supplement	6/9/12 g	dispersed in infant formula	4 wk	Stool frequency; Straining effort and pain; Bifidobacterium genus counts	The FOS group exhibited a higher frequency of softer stools and fewer episodes of straining and/or difficulty passing stools.
[27]	Japan	Constipation	Both	preschool children	23	Placebo (maltose)	1-Kestose	3 g/d		8 wk	Stool frequency; Bifidobacterium genus counts	Three grams of kestose per day for 8 weeks did not affect stool properties, but significantly increased the number of defecations per week; A significant decrease in Intestinibacter, a trend toward increased bifidobacteria.
[24]	Japan	Healthy pregnant woman	Pregnant women	30–33	64	Placebo (sucrose)	FOS	8 g/d	oral	14 wk	Stool frequency	Stool frequency in the FOS group was slightly higher than that in the placebo group
[31]	Basel	Healthy	Both	18–57	16	Placebo syrup (molasses)	yacon syrup	20 g/d (equal to 6.4 g FOS)	syrup	2 wk	Stool frequency;	Stool frequency increased from 1.1 ± 0.1 to 1.3 ± 0.2 times per day and the consistency showed a tendency for softer stools as assessed by a numerical depicted stool protocol.
[34]	Spain	Constipation	Both	2–5	17	Placebo (maltodextrin)	mixture of inulin and FOS	4 g/d	mixed into a dairy product	6 wk	Stool frequency; Stool consistency	About one additional bowel movement per week; Prebiotic inulin-type fructans supplementation improves stool consistency in constipated 2–5-year old children.
[36]	Spain	Diagnosis of gynecological cancer requiring postoperative pelvic RT	Female	>18 60.3 ± 11.8	38	Placebo (maltodextrin)	a mixture of fiber (50 inulin and 50% FOS)	12 g/d	in powder form were dissolved in 200 cc of water	29 d	Stool frequency; Stool consistency	Significantly increased the stool frequency; Improve the consistency of stools in gynecologic cancer patients
[23]	America	Healthy	Both	18–50	98	matching control foods	Oligofructose (OF)	16 g/d	in yogurt and snack bars	8 wk	Stool frequency	Significantly increased the stool frequency; Mean daily symptom intensity scores for flatulence and noises were significantly higher with oligofructose.
[32]	Belgium	Healthy	Both	46.9	20	Placebo (non-carbonated soft drinks without WBE and oligofructose)	Wheat bran extract (WBE)/Oligofructose	30 g/d	preparation	6 wk	Stool frequency; Stool consistency	The bowel habits parameters (defecation frequency and stool consistency) were not modulated by the intake of 30 g/d oligofructose
[22]	China Taiwan	Constipation	men	≥60	5	(regular diet)	FOS	10 g/d	jellies	30 d	Stool frequency;	Significantly increased the defecation frequency, daily stool weight, weight per stool
[28]	Japan	Healthy	Female	18–21	34	Placebo (white colored cellulose pills coated with zein)	FOS	3 g/d	tablet	14 d	Stool frequency; Straining effort and pain	FOS significantly increased the bowel evacuation rate, reduced perception of straining effort and pain.
[33]	UK	Healthy	Both	18–50	38	Placebo (maltodextrin)	mixture of fructan and FOS	5 g/d	purified powder	21 d	Stool frequency; Stool consistency; Bifidobacterium genus counts	Agave fructans are well tolerated in healthy human subjects and increased bifidobacteria and lactobacilli numbers in vitro and in vivo but did not influence other products of fermentation.

## Data Availability

The data presented in this study are available on request from the corresponding authors. The data are not publicly available due to privacy restrictions.
